# Exploiting intrinsic fluctuations to identify model parameters

**DOI:** 10.1049/iet-syb.2014.0010

**Published:** 2015-04-01

**Authors:** Christoph Zimmer, Sven Sahle, Jürgen Pahle

**Affiliations:** ^1^ BIOMS, Heidelberg University Im Neuenheimer Feld 267 69120 Heidelberg Germany; ^2^ BioQuant, Heidelberg University Im Neuenheimer Feld 267 69120 Heidelberg Germany; ^3^ School of Computer Science, Manchester Institute of Biotechnology, The University of Manchester 131 Princess Street Manchester M1 7DN UK

**Keywords:** biochemistry, physiological models, stochastic processes, measurement errors, fluctuations, parameter estimation, model parameter identification, deterministic framework, biochemical system, steady state, transient state, stochastic modelling framework, objective function, immigration‐death model, gene expression, Epo–EpoReceptor interaction, stochastic fluctuations, measurement noise

## Abstract

Parameterisation of kinetic models plays a central role in computational systems biology. Besides the lack of experimental data of high enough quality, some of the biggest challenges here are identification issues. Model parameters can be structurally non‐identifiable because of functional relationships. Noise in measured data is usually considered to be a nuisance for parameter estimation. However, it turns out that intrinsic fluctuations in particle numbers can make parameters identifiable that were previously non‐identifiable. The authors present a method to identify model parameters that are structurally non‐identifiable in a deterministic framework. The method takes time course recordings of biochemical systems in steady state or transient state as input. Often a functional relationship between parameters presents itself by a one‐dimensional manifold in parameter space containing parameter sets of optimal goodness. Although the system's behaviour cannot be distinguished on this manifold in a deterministic framework it might be distinguishable in a stochastic modelling framework. Their method exploits this by using an objective function that includes a measure for fluctuations in particle numbers. They show on three example models, immigration‐death, gene expression and Epo‐EpoReceptor interaction, that this resolves the non‐identifiability even in the case of measurement noise with known amplitude. The method is applied to partially observed recordings of biochemical systems with measurement noise. It is simple to implement and it is usually very fast to compute. This optimisation can be realised in a classical or Bayesian fashion.

## 1 Introduction

Mathematical models of biochemical networks are an indispensable tool in systems biology [1, 2]. They allow us to effectively explore the dynamic behaviour of biochemical reaction networks, thereby, helping greatly with understanding these systems. Even though there are many biological data already available, such as the ones produced by high‐throughput genomic or proteomic measurement techniques and, increasingly, single‐cell recordings [3], there is still a lack of knowledge about most kinetic reaction parameters. These are, however, needed when setting up mathematical models. In practice, unknown parameters are estimated using measured data, for instance, time courses of chemical species’ concentrations in a lab experiment. The parameters in the model are then changed using different approaches such that the simulation is as close to the behaviour of the real system as possible.

Often, systems of ordinary differential equations (ODEs) are used to describe the dynamic processes involved, for example, conversions of chemical substances into each other. In this deterministic framework, problems of non‐identifiability can arise. This means that different sets of parameters can fit the experimental data equally well and estimators are not able to find one unique optimal value for each parameter. The type of non‐identifiability we focus on in this paper is called structural non‐identifiability [4]. Here, there are functional relationships between parameters, meaning that if one parameter value is changed one or several other parameter values can be changed also such that the same system behaviour is retained.

In addition to non‐identifiability issues, noise in experimental measurements creates yet another challenge for parameter estimation. With noise we mean variability of recorded values that is due to causes we do not – or cannot – account or correct for. Examples are fluctuations in the measurement device and in the environment, or differences in size, geometry or protein content of the single cells measured. This noise can make parameter estimation difficult because it adds uncertainty to the measurements.

However, there also exists a different type of noise, namely fluctuations in molecular numbers because of stochastic timings of single discrete reaction events in a biochemical system. This type of noise is called intrinsic noise to distinguish it from other types of noise that are subsumed under the term extrinsic noise [5]. Intrinsic noise cannot be separated from the dynamics of the biochemical system and its amplitude and characteristics are dependent on elementary reaction steps. Therefore these intrinsic fluctuations can reveal more information about the underlying processes than averaged or smoothed recordings. While extrinsic noise reduces the information we have about a system, intrinsic noise can actually increase it. For example, even if a system is in a steady state it can still show fluctuations because of intrinsic stochasticity. These fluctuations act as continued perturbations to the system and can render the reactivity of the system visible. Progress in experimental techniques allows to measure these fluctuations in small numbers of molecules in single cells [6]. On the computational side, simulation of intrinsic noise was pioneered by Gillespie [7] and now a large variety of different simulation algorithms is available [8].

We suggest a simple but practical method to make use of intrinsic fluctuations for identifying model parameters. It is based on an objective function comparing experimental data to simulated data. This method can employ different measures to quantify fluctuations and we study one measure based on differences between consecutive measurements and one based on autocorrelations. While the autocorrelation‐based measure did not perform well in our test cases, using the differences‐based measure allowed us to identify previously (structurally) non‐identifiable parameters in an immigration‐death model, a gene expression model [9] and a model of erythropoietin and Epo receptor interaction [10]. Our method is particularly useful when perturbation experiments cannot easily be made as it allows to identify model parameters from steady state recordings alone, although it is not restricted to steady states.

A related study also makes use of intrinsic fluctuations to estimate parameters [11]. Other methods involve stochastic gradient descent [12], Bayesian techniques [13] or a finite state projection for the solution of the chemical master equation [14]. However, for large systems these computations become very time consuming. Our method focuses on systems that are structurally non‐identifiable with deterministic ODE modelling because of a one‐dimensional (1D) functional relationship of two or more parameters. Using the information about this non‐identifiability in combination with a measure for intrinsic fluctuations allows for a very fast estimation procedure. Correlation‐based measures have also been used in the context of network topology inference, for example, using partial correlation coefficients [15], and for the detection of activity in regulatory links of genes [16].

Furthermore, we consider it an important property of the method presented here that it can be easily implemented in software, thus making the method accessible for a wide range of potential users. In fact, starting from an existing stochastic simulator [17] the method was implemented in only a few lines of Mathematica [18] code.

This paper is structured in Section 2 that explains the modelling framework and where the differences‐based as well as autocorrelation‐based methods are introduced. Section 3 shows the performance of the proposed methods for an immigration‐death model, a gene expression model studied in [9] and a model of erythropoietin and Epo receptor interaction developed by Raue *et al.* [10]. Finally, the results are discussed in Section 4.

## 2 Methods

As an example, consider a reaction system describing an immigration‐death process

(1) $$\begin{array}{c} Ø\;\overset{\theta_{1}}{⟶}\;X\\ X\;\overset{\theta_{2}X}{⟶}\;Ø \end{array}$$

where *X* is a biochemical species, Ø denotes a constant source or sink (empty set of substrates or products in a reaction) and *θ*_1_ and *θ*_2_ are kinetic parameters. In the framework of deterministic modelling we can set up an ODE for this system, with *x* the amount of species *X*

$$\frac{dx}{dt}=\theta_{1}-\theta_{2}x,\;x(0)=x_{0}$$

In general, we obtain an initial value problem that for most realistic biochemical models needs to be numerically integrated. For this simple example, however, the solution is straightforward. Setting the initial value $x_{0}=\frac{\theta_{1}}{\theta_{2}}$ it follows that $\frac{dx}{dt}=0$ and, therefore, $x_{\left(\theta_{1},\theta_{2}\right)}(t)=\frac{\theta_{1}}{\theta_{2}}$, independently of time *t*, that is, the system is in a steady state. This steady state is stable and it is the only one for this system. This means that for each value of the quotient of *θ*_1_ and *θ*_2_ the system shows a certain asymptotic behaviour, which stays the same as long as the kinetic parameters are only changed by the same factor. This property is not unique to the immigration‐death system. Such functional relationships between parameters, quotients or more complicated types, also frequently appear in more realistic models. In the context of parameter estimation for biological systems this is referred to as structural non‐identifiability, a non‐identifiability that cannot be resolved even if perfect data is available. This is in contrast to so‐called practical non‐identifiabilities that are due to lack of data, measurement noise and so on [4].

An alternative for the modelling of networks of chemical reactions is a stochastic approach. This includes, *inter alia*, continuous‐time Markov jump processes [19] and stochastic differential equations (Langevin equations). A very popular simulation algorithm for these types of models was developed by Gillespie [7]. For a review of different stochastic simulation algorithms see Pahle [8]. If the deterministic or stochastic approach is most appropriate depends on properties of the specific model [20]. However, in the limit of very large particle numbers and volumes, such that the concentrations are kept the same, the solutions of the stochastic and the deterministic approaches converge [21].

**Fig. 1:**
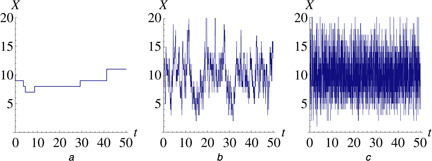
Stochastic realisations of the immigration‐death model *a* This figure illustrates that although the parameters are structurally non‐identifiable in the deterministic framework taking into account intrinsic stochasticity they become identifiable Simulated with the Direct Method [7] in the software Copasi (Version 4.9.43) [17] starting at *x*_0_ = 10 from *t* = 0 to time *t* = 50 with parameter values: Panel a (*θ*_1_, *θ*_2_) = (0.1, 0.02) *b* Panel b (*θ*_1_, *θ*_2_) = (25, 2.5) *c* Panel c (*θ*_1_, *θ*_2_) = (500, 50)

Using the stochastic approach the immigration‐death system can be modelled with a master equation [22] and single instances of the system's behaviour can be computed using a stochastic simulation algorithm such as the Gillespie algorithm [7]. Fig. 1 shows three time courses of the model with different parameter sets that have the same quotient but with very different behaviour. This illustrates that if stochastic trajectories are available instead of just a deterministic steady state it becomes possible to estimate not only the quotient but also the absolute values of both parameters.

In the following, we will assume that the system is non‐identifiable (in the deterministic framework) on a 1D subset Θ of the parameter space because of a functional relationship *ρ* between one and the remaining parameters. This leads to an identical system behaviour of the ODE model for all parameter sets on this 1D subset. With *ϕ* the deterministic solution

(2) $$\phi_{\left(\theta_{1},\rho (\theta_{1})\right)}(t)=\phi_{\left(\theta_{1}^{\prime },\rho (\theta_{1}^{\prime })\right)}(t),\;\text{with}\;\theta_{1},\theta_{1}^{\prime }\in \Theta$$

The functional relationship *ρ* is either known *a priori* or it can be estimated, for example, by fixing *θ*_1_ – which makes the system identifiable – and then estimating the other parameters, that is, *ρ* (*θ*_1_). The full functional relationship *ρ* can then be approximated by performing a scan over a range of values for *θ*_1_ in the subset Θ and using an interpolation scheme. This calculation can be carried out in existing software, such as Copasi [17].

The strategy for identifying *θ*_1_ is to consider random fluctuations not only as a result of extrinsic fluctuations (measurement noise etc.) but also to be caused by inseparable intrinsic stochasticity that can be modelled with Markov jump processes. This intrinsic stochasticity can provide valuable information about the underlying dynamic process, whereas extrinsic stochasticity only decreases the amount of information that can be extracted from measurements.

We suggest to define a 1D non‐identifiability, as described above, as ‘stochastically resolvable’ if

(1) Equation (2) holds in the deterministic (ODE) model for a *ρ* on a parameter subset Θ.
(2) ∃*θ* * such that ∀*θ* ∈ Θ\*θ* *:*E* (*F* (*θ* *)) < *E* (*F* (*θ*)), where *E* (*F* (*θ*)) is the expectation of a suitable objective function *F*.
(3) A suitable objective function *F* is one that quantifies properties of the intrinsic fluctuations both in the experimental data and stochastic simulations, and that represents the distance between these two quantifications.

Please note that this definition does not readily provide a criterion that can be used *a priori* to decide whether a non‐identifiability is stochastically resolvable or not. However, if a particular choice of an objective function leads to a unique minimum on the subset Θ we can *a posteriori* definitely say that there is a stochastically resolvable non‐identitiability present.

A suitable objective function can be of the form *F* (*θ*) = (*D* (*ν*) *−* *D* (*H* (*θ*)))^2^. Here, *D* is a distance measure dependent on the data *ν* quantifying the fluctuations in the system. *H* is simulation results of a stochastic model dependent on parameter set *θ*. The average of the distance measure over *M* simulations will be close to the expectation for large *M*.

For the construction of such an objective function *F* it is important to take into account the limitations of experimental data, for example, measurement errors, small number of data points and large inter‐sample distances. This last point means that not every single reaction event in the system can be observed. Modern measurement techniques [6] can provide molecule numbers on single‐cell level that can be analysed with our method.

In the following, we describe two different simple choices for distance measures *D*. However, the mathematical framework is not dependent on the specific version of the distance measure *D* and other functions can also be used.

### 2.1 Differences‐based measure

Although the deterministic (ODE) model of the system shows identical behaviour for each set of parameters on the subset Θ, Fig. 1 illustrates that stochastic models can show dramatically different behaviours for different parameter sets on the same subset. Intuitively, the reactivity of the system, for example, the average number of reactions per time, changes with changing parameters. This leads to an approach that simply uses the differences between values at subsequent time points of a set of equally spaced measurements.

Now let *ν'* = (*ν'*_1_, *…*, *ν'_n_*) denote the measurements at time points *t*_1_, *…*, *t_n_*. To account for possible mean field dynamics in the system the ODE dynamics *ϕ* (*t*) is subtracted from the measurements

$$\left(\nu_{1},\ldots ,\nu_{n})=(\nu_{1}^{\prime }-\phi (t_{1}),\ldots ,\nu_{n}^{\prime }-\phi (t_{n})\right)$$

Then define

$$D_{\text{diff}}(\nu )=\frac{1}{n-1}\sum_{i=2}^{n}\left|\nu_{i}-\nu_{i-1}\right|$$

Let *H'* ^(*j*)^ (*θ*) = (*H'* (*θ*, *t*_1_), *…*, *H'* (*θ*, *t_n_*)) be the values of the *j* th simulation and *H* ^(*j*)^ (*θ*) = (*H* (*θ*, *t*_1_), …, *H* (*θ*, *t_n_*)) be the values of the *j* th simulation after subtraction of the mean field dynamics. The fitness of a parameter set *θ* is then given by

(3) $$F_{\text{diff}}\left(\theta ,\nu \right)=(D_{\text{diff}}(\nu )-\frac{1}{M}\sum_{\;j=1}^{M}D_{\text{diff}}\;(H^{\left(j\right)}(\theta ))){\;}^{2}$$

with *M* the number of simulations and *θ* = (*θ*_1_, *ρ* (*θ*_1_)) where *ρ* (*θ*_1_) can be determined, given *θ*_1_, by the functional relationship *ρ*. Hence for the situation described above the parameter to be optimised is *θ*_1_. The resulting optimisation problem is

$$\underset{\theta_{1}}{\arg\;\min}\;F_{\text{diff}}\left((\theta_{1},\rho (\theta_{1})),\nu \right)$$

The functional is extended for normally distributed, homoscedastic measurement noise with zero mean by enlarging the parameter vector from *θ* to $\tilde{\theta }=(\theta ,\sigma )$ where *σ* stands for the standard deviation of the measurement error. A simulation $H(\tilde{\theta })$ then means

$$\begin{array}{rl} & H(\tilde{\theta })=(H(\theta ,t_{1})+N(0,\sigma ),\ldots ,H(\theta ,t_{n})+N(0,\sigma )) \end{array}$$

With the variance *σ* unknown, the optimisation problem becomes

$$\underset{\left(\theta_{1},\sigma \right)}{\arg\;\min}\;F_{\text{diff}}\left((\theta_{1},\rho (\theta_{1}),\sigma ),\nu \right)$$

### 2.2 Autocorrelation‐based measure

The autocorrelation function *R* of a stationary stochastic process *x_t_* with *E* [*x_t_*] = *μ* and variance var(*x_t_*) = *σ* ^2^ > 0 depending on time lag Δ is defined as

$$R(\Delta ,x_{t})=\frac{E\left[\left(x_{t}-\mu \right)\left(x_{t+\Delta }-\mu \right)\right]}{\sigma^{2}}$$

Given equally spaced univariate observations from a time series $x=(x_{t_{1}},\ldots ,x_{t_{n}})$, the autocorrelation can be estimated with

$$\hat{R}(k,x)=\frac{1}{(n-k)\sigma^{2}}\sum_{i=1}^{n-k}\;(x_{t_{i}}-\mu )(x_{t_{i+k}}-\mu )$$

where *k* represents the time lag, Δ = *k* (*t*_1_ *−* *t*_0_). If the mean and variance are unknown they can be replaced by the sample estimates.

Assume that *x* is such that $\tilde{k}=\text{min}(k|\hat{R}(k,x)<l)>0$ exists for a level *l*, meaning there is a time lag $\tilde{k}$ where the autocorrelation function is below a user‐defined threshold *l* for the first time. Then define the level‐specific autocorrelation time as

$$D_{\text{act}}(l,x)=(t_{1}-t_{0})\left(\tilde{k}-1+\frac{l-\hat{R}(\tilde{k}-1,x)}{\hat{R}(\tilde{k},x)-\hat{R}(\tilde{k}-1,x)}\right)$$

This is the first crossing of *l* of the autocorrelation function calculated with a linear interpolation between the last value above and the first value below *l*. Although this definition seems straightforward the term autocorrelation time has apparently not been precisely defined in the literature [23]. However, in Gonze *et al.* 2002 [24], the concept of autocorrelation time is used for the investigation of the robustness of circadian rhythms.

Now let *ν* = (*ν*_1_, …, *ν_n_*) denote the measurements at time points *t*_1_, …, *t_n_* and *H* ^(*j*)^ (*θ*) = (*H* (*θ*, *t*_1_), …, *H* (*θ*, *t_n_*)) denote the values of the *j* th simulation. Then the fitness using the autocorrelation *F*_act_ of a parameter *θ* is given by

$$F_{\text{act}}\left(\theta ,\nu \right)={\left(D_{\text{act}}(\nu )-\frac{1}{M}\sum_{\;j=1}^{M}D_{\text{act}}\;\left(H^{\left(j\right)}(\theta )\right)\right)}^{2}$$

with *θ* = (*θ*_1_, *ρ* (*θ*_1_)). The optimisation problem is again

$$\underset{\theta_{1}}{\arg\;\min}\;F_{\text{act}}((\theta_{1},\rho (\theta_{1})),\nu )$$

## 3 Results

### 3.1 Immigration‐death process

First, we study the immigration‐death process [see (1)]. This is an instructive example but it also shows how the method works in the neighbourhood of stable states in much more complicated models. This is because the immigration‐death process resembles a linearisation around a stable steady state and, therefore, the behaviour of this process approximates the behaviour of more complicated processes whenever fluctuations are sufficiently small. In other words, the approximation is good when the linear noise approximation is valid [25–29].

It is often experimentally difficult to observe transient dynamics, primarily when cells cannot easily be synchronised. It can be easier, however, to measure time courses of cells that are in a stable state. This is a scenario that our approach is particularly suited for. In Section 2, we showed that the parameters of the immigration‐death process are structurally non‐identifiable when modelled with ODEs. This situation is not remedied even by investigating cross‐sectional properties of fluctuations, such as marginal variance, as the variance is also only dependent on the quotient of the kinetic parameters $\frac{\theta_{1}}{\theta_{2}}$. To resolve this, we apply our method, namely stochastic modelling in combination with the calculations described in Section 2. The subset Θ of the parameter space is $\{(\theta_{1},\theta_{2})|\frac{\theta_{1}}{\theta_{2}}=\alpha \}$, with a *α* > 0, and hence the function *ρ* takes the form $\rho (\theta_{1})=\frac{\theta_{1}}{\alpha }$, or equivalently *ρ* (*θ*_2_) = *θ*_2_ *α*.

The argumentation holds for the case that the initial value is unknown if time course measurements are only taken from the stable steady state period. Thus, the subset on which the parameters are non‐identifiable using ODE techniques is $\{\theta |\frac{\theta_{1}}{\theta_{2}}=x^{s}\}$ with *x* ^s^ being the steady state of the system. Therefore it is possible to determine the function *ρ* simply by using the steady state: $\rho (\theta_{1})=\frac{\theta_{1}}{x^{s}}$, or equivalently *ρ* (*θ*_2_) = *θ*_2_ *x* ^s^. In more complicated systems the functional relationship between parameters, that is, the manifold on which parameters are non‐identifiable, has to be found by scanning one parameter and performing a series of optimisations for a deterministic objective function that is dependent on the remaining parameters in the set. For the following analysis the function $\rho (\theta_{1})=\frac{\theta_{1}}{x^{s}}$ is used.

The location of the system's steady state can be estimated from data simply with $x^{s}≃\bar{\nu }=\frac{1}{n}\sum_{i=1}^{n}\;\nu_{i}$. However, if an initial condition is known it should be used instead to eliminate one source of uncertainty.

#### 3.1.1 Differences‐based measure

**Fig. 2:**
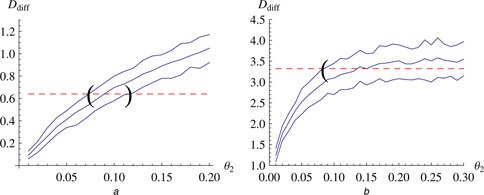
Identification of the previously non‐identifiable parameters of the immigration‐death process with the differences‐based objective function *a* Values of the measure *D*_diff_ (*y* ‐axis) against kinetic parameter *θ*_2_ (*x* ‐axis) Panel a: a realisation of the stochastic immigration‐death model with (*θ*_1_, *θ*_2_) = (1, 0.1) and 100 samples over 50 s was generated and used as input data Dashed line represents the value of measure *D*_diff_ calculated on this data. A range of values for parameter *θ*_2_ was scanned and *D*_diff_ evaluated on 100 simulated stochastic time series for each value Solid lines show estimates of the corresponding 10%‐quantile, mean and 90%‐quantile Intersection of the 10%‐quantile and the 90%‐quantile with the *D*_diff_ value from the data are marked with brackets This represents a confidence interval for the minimum of the objective function *F*_diff_, for example, the estimate for *θ*_2_ *b* Panel b: same calculation as in panel a but with a different sampling of the process (100 samples over 1400 s)

We generated a realisation of the stochastic version of the immigration‐death model with parameters (*θ*_1_, *θ*_2_) = (1, 0.1) using the Direct Method in Copasi (Version 4.9.43) [17] with 100 samples over a simulated time interval of 50 s. This time course was regarded as input data *ν* on which we calculated the differences‐based measure *D*_diff_. The dashed line in Fig. 2, panel a, represents the value of *D*_diff_ (*ν*). We then systematically scanned a range of values for parameter *θ*_2_, computed 100 stochastic simulations for each value and evaluated the objective function *D*_diff_ on the simulated time courses. The 10%‐quantile, mean, 90%‐quantile of these 100 simulations are used to create linearly interpolated lines. These are plotted in Fig. 2, panel a, as solid lines. The intersection of the 10%‐quantile and the 90%‐quantile of the simulations with the *D*_diff_ value from the data are marked with brackets. This represents a confidence interval for the minimum of the objective function *F*_diff_ (3). The existence of such a minimum indicates that the previously structurally non‐identifiable parameter *θ*_2_ has become identifiable, and the width of the interval shows how accurately it can be identified.

The true value of *θ*_2_ (0.1) lies within the interval even though we used a comparatively modest number of 100 observed data points. Also, the 10%‐quantile, mean, 90%‐quantile lines look ragged because we only used 100 simulations for their estimation. This could be improved by increasing the number of simulations for each parameter value. Finally, we did not assume the value of the steady state to be known. Instead we used the sample mean of the data as an estimate, $\bar{\nu }=10.5$, while the true steady‐state value of the system was *x* ^s^ = 10. Nevertheless, using our approach we could resolve the issue of structural non‐identifiability.

The differences‐based objective function *F*_diff_ can be optimised, for example, with a black‐box optimiser such as the particle swarm algorithm [30], to obtain a point estimate for *θ*_2_. For these calculations, we have used a particle swarm algorithm implemented in Mathematica (Version 9.0.1.0) [18] with 10 particles, 25 generations and 200 simulations for the evaluation of the objective function. The simulations are performed in Copasi (Version 4.9.43) [17] called from a Python script. The range for the optimisation is [0, 1]. For the data set underlying Fig. 2 and assuming that the true steady‐state value is unknown this yields an estimate of $\left(\hat{\theta_{1}},\hat{\theta_{2}})=(0.97,0.092\right)$.

One important point that touches upon optimal experimental design, is the choice of the inter‐sample distance, for example, how frequently the system is measured. If the inter‐sample distance is too large or the reactivity, for example, the average number of reactions per time unit in the system, is very high it might happen that two succeeding observations are independent and the autocorrelation time is smaller than the inter‐sample interval.

In Fig. 2 (panel b), the autocorrelation time (4.5 s, level *l* = 0.6) is smaller than the inter‐sample distance (25 s). Varying the parameter, for example, from *θ*_2_ = 0.15 to *θ*_2_ = 0.3 still changes the reactivity of the system but the distance‐based measure *D*_diff_ does not record this because of the too large inter‐sample distance. If the inter‐sample distance is reduced, as in Fig. 2 (panel a) the autocorrelation time is larger than the inter‐sample distance (0.5 s) and the parameter is identifiable.

On the other hand, if the average number of reactions per time is very low (data not shown), almost no reaction happens. A modest change of the parameter will lead to a situation where still almost no reaction happens. Again the value of *D*_diff_ does not record the change of the parameter and the parameter remains non‐identifiable. An increase of the inter‐sample distance can resolve this problem.

Another important point is how measurement errors affect the parameter estimation. In the following we study this aspect for the differences‐based measure using simulation studies. The reason for performing simulation studies is that each estimation obviously depends on the stochasticity in the data. Hence, to reliably assess the performance of an estimator it is necessary to carry out the estimation for many different data sets and analyse the average behaviour of the estimator. We would like to note that this does not mean that many data sets are necessary for an estimation. Each estimation is always performed with one single time course.

Fifty stochastic data sets were generated from the immigration‐death model with the parameter set (*θ*_1_, *θ*_2_) = (1, 0.1) and the initial value *ν* ^(0)^ = 10 using the Direct Method [7] in the software Copasi (Version 4.9.43) [17], each with 100 samples over a simulated time of 50 s. Normally distributed measurement noise was added to each observation with different standard deviations. The differences‐based functional is then used to estimate the parameter *θ*_2_ from the noisy time series. The parameter *θ*_2_ is obtained using the function *ρ* with the mean $\bar{\nu }$ as the estimate for the steady‐state value. Table 1 shows the results under the assumption that the amplitude of the measurement noise, *σ* ^2^, is known. An additive error of standard deviation 1 corresponds to a relative error of 10% as the steady state of the system is 10. Our method can estimate the previously non‐identifiable parameter in these data sets with measurement noise.

**Table 1:** Statistics of estimation results with measurement noise of known standard deviation

	True parameter value	Estimation results	Average relative error, %
exact measurements			
*θ*_1_	1.0	1.03	19
*θ*_2_	0.1	0.103	13
noise with standard deviation 0.01			
*θ*_1_	1.0	1.03	19
*θ*_2_	0.1	0.103	13
noise with standard deviation 0.1			
*θ*_1_	1.0	1.03	22
*θ*_2_	0.1	0.102	15
noise with standard deviation 0.25			
*θ*_1_	1.0	1.04	18
*θ*_2_	0.1	0.104	15
noise with standard deviation 0.5			
*θ*_1_	1.0	1.03	23
*θ*_2_	0.1	0.103	20
noise with standard deviation 1.0			
*θ*_1_	1.0	1.03	46
*θ*_2_	0.1	0.106	47

For each measurement noise scenario 50 data sets were simulated using the Direct Method in Copasi (Version 4.9.43) [17] with 100 samples, Δ*t* = 0.5, (*θ*_1_, *θ*_2_) = (1, 0.1) and *ν*_0_ = 10. For each of the 50 data sets the parameter is estimated using the differences‐based functional. For different noise levels the table gives the true parameter value (column 2), the mean of the 50 estimation results (column 3) and the average relative error (column 4).

Table 2 shows that without knowledge of the standard deviation of the measurement noise the estimation accuracy decreases dramatically, in particular the estimation of the measurement noise level is problematic. The optimisation is again performed with a particle swarm algorithm with 20 particles and 100 iterations and 200 simulations for each evaluation of the objective function on a range of ([0, 1], [0.001, 1]).

**Table 2:** Statistics of estimation results with measurement noise of unknown standard deviation

	True parameter value	Estimation results	Average relative error, %
noise with standard deviation 0.01			
*θ*_2_	0.1	0.083	27
*σ*	0.01	0.151	1409

50 data sets were simulated using the Direct Method in Copasi (Version 4.9.43) [17] with 100 samples, Δ*t* = 0.5, (*θ*_1_, *θ*_2_) = (1, 0.1) and *ν*_0_ = 10. Measurement noise is added to the data. Then these data sets are used to estimate parameter *θ*_2_ with the differences‐based functional. The standard deviation of the measurement noise is assumed not to be known and must therefore be estimated as well. The table shows the estimated parameter (column 1), its true value (column 2) and the mean (column 3) as well as the average relative error of the 50 estimates (column 4).

**Fig. 3:**
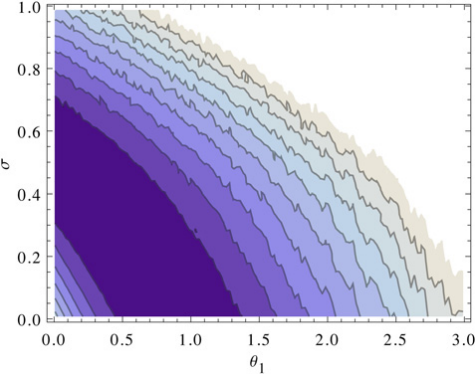
Higher measurement noise leads to an increase in the differences‐based measure Differences‐based objective function and estimation of noise level One time series is simulated with 100 samples over 50 sec and *ν*_0_ = 10 for (*θ*_1_, *θ*_2_) = (1, 0.1) without measurement noise Differences‐based objective function is then evaluated in dependence of *θ*_1_ and *σ* Function *ρ* is of the form $\rho (\theta_{1})=\frac{\theta_{1}}{10}$ Dark colour stands for low objective function values, bright colour stands for high objective function values Plot shows a valley, in which the objective function value remains constant for increasing *σ* and decreasing *θ*_1_

A higher measurement noise leads to an increase in the differences‐based measure and can therefore compensate for a lower reactivity because of lower values of the kinetic parameters. This is illustrated in Fig. 3. Strictly speaking, this means that the parameters remain structurally non‐identifiable as there is no unique minimum of the objective function. Nevertheless, the situation has improved because the region where the parameters are non‐identifiable is now bounded. The parameter values have to be larger than zero as they represent the reaction rates of irreversible reactions. The valley in the landscape of objective function values in Fig. 3 shows that even for the case of zero measurement noise parameter *θ*_1_ cannot be larger than about 1.5. If the amplitude of the measurement noise is known the parameter becomes completely identifiable again. This illustrates that information on the amplitude of measurement noise can be very valuable, although in many practical cases reliable values are notoriously difficult to obtain.

#### 3.1.2 Autocorrelation‐based measure

**Fig. 4:**
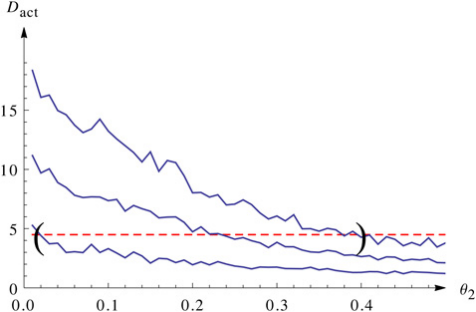
Identification of the previously non‐identifiable parameters of the immigration‐death process with the autocorrelation‐based objective function A realisation with 100 samples over 50 s of the immigration‐death model is simulated with (*θ*_1_, *θ*_2_) = (1, 0.1) This is regarded as input data *ν* Dashed line represents the value of the autocorrelation‐based measure *D*_act_ (*ν*) For a range of values for parameter *θ*_2_ the autocorrelation‐based objective function is evaluated on 100 simulations with a level *l* = 0.6 that seemed to be a robust choice 10%‐quantile, mean and 90%‐quantile of these 100 simulations for each value are used to create linear interpolation lines for the 10%‐quantile, mean and 90%‐quantile These are plotted with solid lines

We repeated the analysis that is shown in Fig. 2 with the autocorrelation‐based measure instead of the differences‐based one. As can be seen in Fig. 4 in this case the autocorrelation‐based measure did not allow a satisfactory identification of the parameters. The 10%‐quantile line of the simulations crosses the line calculated from the input data already at a very low value. Therefore we cannot give a lower bound for the parameter value that is considerably better than the physiological limit at zero. Also, the 90%‐quantile line crosses the line calculated from the data only at relatively high values for the parameter *θ*_2_ meaning that the upper bound is also only a very loose one.

Owing to this poor performance of the autocorrelation‐based measure we focussed on using the differences‐based measure for analysing a more realistic model in the next section.

### 3.2 Single gene expression model

Here, we apply our method to a model of gene expression that was already studied in [9]. The model consists of four reactions

$$\begin{array}{c} Ø\;\overset{k_{r}}{⟶}\;\text{mRNA}\\ \text{mRNA}\;\overset{k_{p}\text{mRNA}}{⟶}\;\text{Pro}+\text{mRNA}\\ \text{mRNA}\;\overset{\gamma_{r}\text{mRNA}}{⟶}\;Ø\\ \text{Pro}\;\overset{\gamma_{p}\text{Pro}}{⟶}Ø \end{array}$$

with true parameter values *k*_r_ = 20, *k*_p_ = 10, *γ*_r_ = 1.2, *γ*_p_ = 0.7 and initial conditions mRNA(0) = mRNA_0_, Pro(0) = Pro_0_. The ODE interpretation reads as follows

(4) $$\begin{array}{rl} \frac{d}{dt}\text{mRNA}(t) & =k_{r}-\gamma_{r}\;\text{mRNA}(t)\\ \frac{d}{dt}\text{Pro}(t) & =k_{p}\;\text{mRNA}(t)-\gamma_{p}\;\text{Pro}(t) \end{array}$$

The deterministic steady state of the system is (mRNA, Pro) = (16.7, 238.1). This corresponds to parameter set 3 in the supplementary information of [9] with a slow protein degradation rate *γ*_p_ and a low transcription/tran^slation ratio. Using perturbation experiments and a parameter estimation for deterministic systems such as least squares it is possible to determine *γ*_r_ and *γ*_p_ plus a functional relation between *k*_r_ and *k*_p_. With this functional relationship and the differences‐based functional it is possible to estimate the absolute value of *k*_r_ and *k*_p_. This can be done with either data from a perturbation experiment or data from a single steady‐state experiment (see Table 3).

**Table 3:** Statistics of estimation results for the gene expression model

	True parameter value	Estimation results	Average relative error, %
perturbation experiment			
*k_r_*	20	20.5	26
steady‐state data only			
*k_r_*	20	20.1	19

Fifty data sets are stochastically simulated with 50 samples, inter‐sample interval Δ*t* = 0.3 and parameters *k*_r_ = 20, *k*_p_ = 10, *γ*_r_ = 1.2, *γ_p_* = 0.7. These data sets are used to obtain estimates for the previously non‐identifiable parameters *k*_r_ and *k*_p_ with the differences‐based functional in a range of [0.1, 100] for the optimisation. The table shows the estimated parameter (column 1), its true value (column 2) and the mean of the estimations (column 3) as well as the average relative error of the 50 estimates (column 4).

Our method was therefore able to reliably estimate the previously non‐identifiable parameter *k*_r_ even from single measurements of the system in steady state and without calculating more involved quantities like the Fisher information and so on.

### 3.3 Epo receptor model

The third example is a model of erythropoietin (Epo) and Epo receptor (EpoR) interaction due to Raue *et al.* [10] with the following differential equations system

(5) $$\begin{array}{rl} \frac{d}{dt}\text{Epo}(t) & =-k_{\text{on}}\text{Epo}(t)\text{Epo}R(t)+k_{\text{on}}k_{D}\text{Epo}\text{Epo}R(t)+k_{\text{ex}}\text{Epo}\text{Epo}R_{i}(t)\\ \frac{d}{dt}\text{Epo}R(t) & =-k_{\text{on}}\text{Epo}(t)\text{Epo}R(t)+k_{\text{on}}k_{D}\text{Epo}\text{Epo}R(t)+k_{t}B_{\text{max}}\\ & \;-k_{t}\text{Epo}R(t)+k_{\text{ex}}\text{Epo}\text{Epo}R_{i}(t)\\ \frac{d}{dt}\text{Epo}\text{Epo}R(t) & =k_{\text{on}}\text{Epo}(t)\text{Epo}R(t)-k_{\text{on}}k_{D}\text{Epo}\text{Epo}R(t)-k_{e}\text{Epo}\text{Epo}R(t)\\ \frac{d}{dt}\text{Epo}\text{Epo}R_{i}(t) & =k_{e}\text{Epo}\text{Epo}R(t)-k_{\text{ex}}\text{Epo}\text{Epo}R_{i}(t)\\ & \;-k_{\text{di}}\text{Epo}\text{Epo}R_{i}(t)-k_{\text{de}}\text{Epo}\text{Epo}R_{i}(t)\\ \frac{d}{dt}{\text{Epo}}_{i}(t) & =k_{\text{di}}\text{Epo}\text{Epo}R_{i}(t)\\ \frac{d}{dt}{\text{Epo}}_{e}(t) & =k_{\text{de}}\text{Epo}\text{Epo}R_{i}(t) \end{array}$$

The model describes the processes taking place at the receptor level in Epo signalling. Epo binds to its receptor EpoR on erythroid progenitor cells triggering cellular responses such as proliferation or differentiation via downstream signalling pathways, for example, the JAK2‐STAT5 pathway, that are not part of the model. The variables in the model represent concentrations of extracellular Epo, EpoR, Epo and Epo receptor complex (EpoEpoR), internalised complex (EpoEpoR_i_), degraded internalised Epo (Epo_i_) and degraded extracellular Epo (Epo_e_). Refer to [10, 31] for details about the model.

Measurements were taken of

$$\begin{array}{rl} & y_{1}=\text{scale}(\text{Epo}(t)+{\text{Epo}}_{e}(t))\\ & \text{and}\\ & y_{2}=\text{scale}\times \text{EpoEpoR}(t) \end{array}$$

with the conversion factor scale [10]. The model is structurally non‐identifiable [10]. However, by fixing one of the non‐identifiable parameters we observe that the subset of the parameter space containing the non‐identifiable parameters is 1D. This means that the model becomes identifiable if one parameter is fixed.

In the following, we assume that the true parameters are

(6) $$\begin{array}{c} \begin{array}{rl} \left(B_{\text{max}},k_{\text{on}},k_{D},\text{scale},\text{Epo}(0)\right) & =(170,0.017,13.2,9.12,300)\\ \text{and}\\ \left(k_{t},k_{e},k_{\text{ex}},k_{\text{di}},k_{\text{de}}\right) & =(0.17,0.3,0.087,0.065,0.15),\\ \left(\text{Epo}R(0),\text{Epo}\text{Epo}R(0),\text{Epo}\text{Epo}R_{i}(0),{\text{Epo}}_{i}(0),{\text{Epo}}_{e}(0)\right) & =(B_{\text{max}},0,0,0,0). \end{array} \end{array}$$

**Fig. 5:**
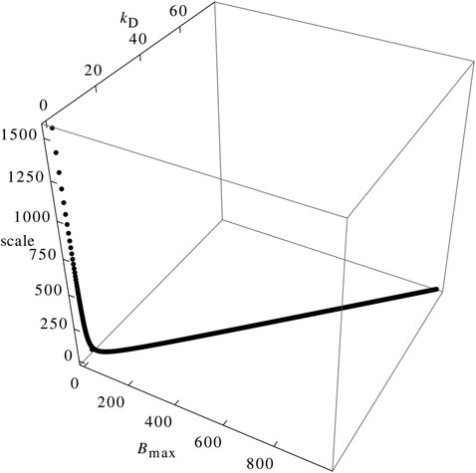
Non‐identifiable subset of the parameter space This plot shows the non‐identifiable subset of the parameter space in the Epo receptor model *B*_max_ is fixed over a range of values and the parameter vector (*k*_on_, *k*_D_, scale and Epo(0)) is optimised with a deterministic trajectory, calculated from the ODE, as a reference

To identify the relationship between the non‐identifiable parameters *B*_max_ and (*k*_on_, *k*_D_, scale and Epo(0)) we first calculated a deterministic trajectory from the ODE model (5) as a reference. Then we fixed *B*_max_ over a range of values and optimised (*k*_on_, *k*_D_, scale and Epo(0)) for each value. Fig. 5 shows a plot of the resulting subset depending on the parameters *B*_max_, scale and *k*_D_. As the subset is 1D our method can be used to estimate the non‐identifiable parameters.

We stochastically simulated 50 time courses from the Epo receptor model using the Direct Method in the software Copasi (Version 4.9.43) [17]. These time courses are then used as data to estimate the parameter *B*_max_ using the differences‐based functional in (3). The optimisation was performed with a particle swarm algorithm implemented in Mathematica. Ten particles and 25 iterations were used and 200 simulations for each evaluation of the objective function on a range of [100, 1000]. The means of the estimation results are given in Table 4 together with their relative error. The table shows that the previously non‐identifiable parameter becomes identifiable in cases of small measurement noise. The other parameters *k*_on_, *k*_D_, scale and Epo(0) can be calculated with the knowledge of *B*_max_ from the parameterised function.

**Table 4:** Statistics of estimation results for the Epo receptor model

	True parameter value	Estimation results	Average relative error, %
no noise			
*B*_max_	170	170	9
noise with standard deviation 5			
*B*_max_	170	171	10
noise with standard deviation 10			
*B*_max_	170	175	12
noise with standard deviation 25			
*B*_max_	170	191	36
noise with standard deviation 50			
*B*_max_	170	352	132
relative noise 1%			
*B*_max_	170	171	12
relative noise 5%			
*B*_max_	170	333	126

Fifty data sets are stochastically simulated with 160 samples, inter‐sample interval Δ*t* = 0.1 and the parameter set given in (6). Measurement noise with known standard deviation is then added to the data. These data sets are used to obtain estimates for the previously non‐identifiable parameter *B*_max_ with the differences‐based functional in a range of [100, 1000] for the optimisation. The table shows the estimated parameter (column 1), its true value (column 2) and the mean (column 3) as well as the average relative error of the 50 estimates (column 4).

**Fig. 6:**
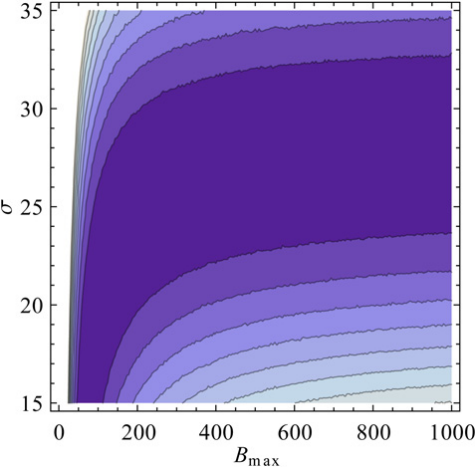
Knowing the magnitude of the measurement error makes the system completely identifiable Differences‐based objective function in dependence on *B*_max_ and noise level One time course is stochastically simulated with 160 samples over 16 sec and the parameters given in (6) Differences‐based objective function values are calculated in dependence of *B*_max_ and measurement noise standard deviation *σ* Dark colour: low function value Bright colour: high function value

If the magnitude of the measurement noise is not known, our method makes the previously structurally non‐identifiable parameter set practically non‐identifiable. This means that the estimation will be better with more data, effectively eliminating measurement errors. In fact, in this specific case eliminating measurement errors by measuring more data is not even necessary. Already knowing just the magnitude of the error makes the system completely identifiable (see Fig. 6).

If only univariate measurements of *y*_1_ are available the estimation is still possible. For the scenario described in Table 4 without measurement noise the mean of the estimation from *y*_1_ alone is 169 for *B*_max_ with an average relative error of 11%. We used a particle swarm method to find the optimum within the limits [100, 1000].

## 4 Discussion and conclusion

Deterministic models based on ODEs are often used in systems biology to describe dynamic processes. Techniques for the estimation of parameters in these systems are highly developed [32, 33]. Nevertheless limitations in measurement techniques or the structure of the model itself can lead to identifiability problems, such as practical and structural non‐identifiabilities, respectively. This paper presents a simple, yet practical, method for the identification of model parameters making use of intrinsic fluctuations. Two measures are introduced as objective functions for parameter estimation. Both measures search for a parameter set that produces intrinsic fluctuations fitting best to the experimental data – in this study the input data was also generated by simulation for a fairer comparison. We tested our method on a simple immigration‐death system, on a model of gene expression and on a more realistic model of Epo receptor signalling.

The autocorrelation‐based method uses the autocorrelation time of the system in its objective function. Its performance was not as good as the difference‐based measure (Fig. 4) on the immigration‐death model we tested it on, and so we did not use it to analyse the more realistic models later on. Nevertheless, it also yielded a confidence interval, albeit a fairly large one, for the previously non‐identifiable parameter. A possible reason for the weaker performance of the autocorrelation‐based method is the fact that the calculation of an autocorrelation time for a time series with limited length and sampling rate is not very accurate, especially without prior knowledge about the underlying dynamics.

Another complication that might arise with the autocorrelation‐based measure is in systems that are not stationary and where, therefore, an autocorrelation cannot be easily defined.

The differences‐based measure, on the other hand, allowed to fully identify the previously non‐identifiable parameters assuming that the amplitude of the measurement noise is known. This assumption is also regularly made in related studies (see, e.g. [4]). Even if the measurement noise is unknown, our method can be very useful. In the immigration‐death system it was able to change a structural non‐identifiability into a practical non‐identifiability. This means that there was no single optimum in the estimation, but an interval of values could be given with equally good fits.

Compared with other methods that also make use of intrinsic fluctuations [12–14] the presented method is computationally fast because the optimisation problem is 1D as information on the structure of the non‐identifiability manifold is used. This functional relationship can be estimated by performing estimation procedures on a deterministic ODE model of the system fixing one of the non‐identifiable parameters over a range of different values and recording the estimation results of the remaining parameters. An interpolation over these discrete values yields the relationship.

Our method is particularly suitable to study time courses of noisy steady states. This is especially important, for example, when it is difficult to measure initial concentrations, when the cells cannot be synchronised or when the system cannot easily be perturbed. We would like to emphasise that it is not necessary to have dense measurements in the sense that every single reaction event has to be measured in order to account for the stochasticity.

In this paper, we exclusively used equally spaced measurements. An extension to non‐equally spaced measurements should be possible with the introduction of weights.

The (equally spaced) time step between measurements has to be both smaller than the autocorrelation time of the system (Fig. 2) and long enough that a number of reactions takes place in between. An experimental setup not chosen carefully enough might lead to an almost or completely flat landscape and therefore not allow resolving non‐identifiabilities. This question of optimal measurement intervals and how they can be determined for our method falls under the broader category of optimal experimental design and should be investigated in future studies.

Further research could also provide a correction term to correct for a possible bias introduced by high measurement noise. In the case of the Epo receptor signalling model with measurement noise we observed such a bias. Studying the size of the bias depending on the measurement noise it should be possible to introduce a correction term.

The autocorrelation‐based method had a poorer performance on the immigration‐death system compared with the differences‐based measure. It might be interesting to find out if this is a general property or if there are systems where the autocorrelation‐based measure performs better.

Systems with 1D non‐identifiabilities, which we focused on in this paper, are certainly of practical relevance [10]. For systems that have a non‐identifiability that is more than 1D this might become resolvable if multivariate recordings are available.

Finally, it could be useful to have a criterion that, *a priori*, indicates whether a non‐identifiability is stochastically resolvable or not. It seems plausible that the validity of the linear noise approximation is a necessary condition as in systems, which do not satisfy it, other effects than the reactivity might influence the differences‐based functional. However, there exist methods that can provide correction terms in cases where the linear noise approximation fails [28, 29], for example, in systems that have highly non‐linear kinetics and very low particle numbers. Integrating these methods with our approach is possible and might even allow the treatment of systems where a simple linear noise approximation is problematic. In any case, after the calculations described in this paper have been carried out it can be determined whether the non‐identifiability could be removed.
